# Associations between parental psychological control and negative emotions in early adolescents: a moderated mediation model

**DOI:** 10.3389/fpsyg.2025.1618832

**Published:** 2025-09-17

**Authors:** Min Zhu, Tao Tu

**Affiliations:** ^1^Department of Mental Health Center, Anhui Technical College of Mechanical and Electrical Engineering, Wuhu, China; ^2^Faculty of Education, Guangxi Normal University, Guilin, China

**Keywords:** parental psychological control, peer rejection, aggressive behavior, awe of life, preadolescence

## Abstract

**Introduction:**

Previous research on negative emotions has primarily focused on middle and high school students. Based on the cumulative risk model framework, this study targeting early adolescents integrates parental psychological control, peer rejection and aggressive behavior to investigate their associations with negative emotions, while also examining the protective moderation of awe of life.

**Methods:**

In this cross-sectional study, a total of 1,163 early adolescents (M_age_ = 11.28 ± 0.80 years) completed self-report measures: the Parental Psychological Control Scale, Peer Rejection Scale, Aggressive Behavior Scale, and Awe of Life Scale. A moderated mediation model was tested to examine the mechanisms underlying the association between parental psychological control and negative emotions.

**Results:**

(1) Parental psychological control was significantly positively correlated with peer rejection, aggressive behavior, and negative emotions. (2) Parental psychological control showed a direct positive association with negative emotions (β = 0.179, *p* < 0.001), accounting for 31.57% of the total association. (3) Parental psychological control was associated with negative emotions through the serial mediation of peer rejection and aggressive behavior. The mediating pathways via peer rejection (β = 0.144, *p* < 0.001), aggressive behavior (β = 0.124, *p* < 0.001), and the serial mediation pathway (β = 0.120, *p* < 0.001) accounted for 25.40%, 21.87%, and 21.16% of the total association, respectively. (4) Awe of life significantly moderated only the direct association, with this association being more pronounced under low levels of awe.

**Discussion:**

The chained accumulation of family, peer, and individual risk factors constitutes a core mechanism underlying negative emotions, with parental psychological control emerging as the primary influencing factor. Awe of life, meanwhile, provides buffering protection by moderating the direct pathway. Systemic disruption of this risk accumulation process can be achieved by reducing parental psychological control, improving peer relationships, and cultivating awe of life. These insights offer actionable strategies for families and educators to design targeted interventions aimed at enhancing mental health in early adolescence.

## 1 Introduction

According to World Health Organization (WHO) estimates, approximately 50% of mental health conditions first emerge during adolescence. A meta-analysis of 191 global studies (total *N* = 1,389,447) reported pooled prevalence rates of 31% for depressive symptoms, 31% for anxiety symptoms, and 42% for sleep disturbances among children and adolescents (Deng et al., 2023). (Tang et al. 2020) conducted an online survey of 4,342 primary and secondary school students in Shanghai, China, reporting prevalence rates of 19.7% for depression, 24.9% for anxiety, and 15.2% for stress. Collectively, these findings indicate a deterioration in adolescent mental health that is now recognized as a global crisis (Lawrance, 2024), alongside a discernible trend toward earlier onset ages (Chen, 2023; Hu et al., 2024). (Henry and Crawford 2005) demonstrated that depression, anxiety, and stress constitute the core measurable dimensions of general psychological distress, with their integrated measurement showing robust validity. Building on this framework, the present study consolidates these three dimensions into the “negative emotions” construct, which serves as an operational metric for mental health assessment.

However, current research on adolescent mental health predominantly focuses on middle and high school students (Roulston et al., 2025; Pecora et al., 2025; Karhina et al., 2023), while largely overlooking upper elementary school students—a critical developmental transition period. The mismatch between rapid biopsychosocial changes (Blakemore et al., 2010; Dehestani et al., 2023) and insufficient cognitive-emotional preparedness (Niu et al., 2023) heightens vulnerability to psychological distress. Consequently, enhancing research on negative emotions in this cohort not only facilitates early identification of emerging emotional disturbances, but also significantly advances our understanding of the continuity of emotional development throughout adolescence.

Parenting styles significantly influence children's internalizing and externalizing symptoms (Lorence et al., 2019). Parental psychological control refers to intrusive strategies that manipulate children's emotions, cognitions, and self-worth to regulate behavior (Barber and Harmon, 2002). These strategies include guilt induction, love withdrawal, and ignoring children's needs. Self-Determination Theory (Deci and Ryan, 2000) points that autonomy, competence and relatedness are innate basic psychological needs in humans. By ignoring children's emotional and psychological needs, parental psychological control undermines the formation and development of their autonomy (Chyung et al., 2021). Extensive empirical research consistently demonstrates that higher levels of parental psychological control predicts multiple internalizing problems in adolescents, including anxiety, depression, loneliness, and social withdrawal (Falcone et al., 2024; Chyung et al., 2021; Pinquart, 2017; Lee et al., 2015; Barber and Harmon, 2002; Yap and Jorm, 2015).

Social Learning Theory (Bandura, 1977) points out that individuals acquire new behaviors and skills through observing and imitating others. Parental rearing practices significantly shape children's behavioral development (Grusec, 2011). Children chronically exposed to parental psychological control internalize this pattern and apply it to their interpersonal interactions, subtly coercing their peers into meeting their demands (Chen and Cheng, 2019), including social exclusion, attention withdrawal, and instrumental friendship manipulation (Crick and Grotpeter, 1995). These behaviors contribute to strained peer relationships and may elicit peer rejection.

Peer rejection is defined as active dislike by peers, which is typically hurtful (Beeson et al., 2020; Gifford-Smith and Brownell, 2003). Adolescents actively form their identities, decreasing parental dependence and increasingly seeking support from their peers (Roach, 2018). Peer support is a protective factor against suicide, depression, anxiety, and stress in adolescents, and is positively associated with mental health, self-esteem, and optimism. In fact, peer rejection frequently occurs. When adolescents experience peer rejection, they exhibit emotional distress (Masten et al., 2009) and develop a range of internalizing and externalizing problems (Loparo et al., 2023), including anxiety, loneliness, social withdrawal, depression, anger, hostility, and aggression (Beeson et al., 2020; Zimmer-Gembeck et al., 2009; Dodge et al., 2003), with such effects demonstrating enduring persistence (Ha et al., 2019).

(Cui et al. 2014) pointed out that parental psychological control significantly predicts aggressive behavior, including relational and physical aggression (Nelson et al., 2013). (Kuppens et al. 2009) found that parental psychological control was positively correlated with relational aggression in children aged 8–10 years. Longitudinal studies confirm that parental psychological control heightens aggressive behavior over time, which in turn is strongly associated with depression (Zhang and Zhang, 2023) and anxiety (Chung et al., 2019).

Peer relationships shape the development of children's behavioral tendencies (e.g., aggression, social withdrawal) (Coie et al., 1992; Gazelle and Ladd, 2003), with adverse peer relationships significantly predicting adolescent problem behaviors (Hinnant et al., 2015; Zhu et al., 2017). (Yue and Zhang 2023) verified that peer rejection exacerbates adolescents' aggression and rule-breaking behaviors (Janssens et al., 2017). Longitudinal studies further reveal that peer rejection during elementary school predicts externalizing behaviors including physical aggression and delinquency in middle school (Laird et al., 2001).

Awe is a complex emotional experience that arises when individuals encounter phenomena that transcend their existing cognitive frameworks (Liao et al., 2024; Keltner and Haidt, 2003). As a positive social emotion (Lin et al., 2025), awe enhances social functioning (Stellar et al., 2017), fosters positive mindsets (Stellar et al., 2018), and cultivates an optimistic life attitude (Yuan et al., 2024). Awe of life refers to an individual's reverential emotions and attitudes toward life, stemming from perceptions of life's miracles, power, fragility, and mortality, thereby evoking a sense of both profound respect and trepidation (Lin et al., 2024). Awe of life has been shown to enhance individuals' quality of life and mitigate negative emotions (Sun et al., 2023). However, research indicates that approximately 22.3% of Chinese adolescents reported suicidal thoughts during the past 12 months (Luo et al., 2022). Leveraging the moderating role of awe of life in mitigating negative emotions holds significant implications for strengthening life education and promoting mental health among adolescents.

### 1.1 The present study

Despite extensive research on parental psychological control, peer relationships, aggressive behavior, and negative emotions as isolated domains, no study to date has comprehensively integrated family, peer, and individual behavioral factors into a unified framework to elucidate their systemic pathways underlying negative emotions. Guided by the Cumulative Risk Model (Evans et al., 2013) framework, this study examines the concurrent associations between multiple risk factors and negative emotions by quantifying their relative contributions, and further investigates the moderating role of awe of life, which provides both theoretical foundations and empirical evidence for alleviating emotional distress in early adolescents.

**Figure 1:**
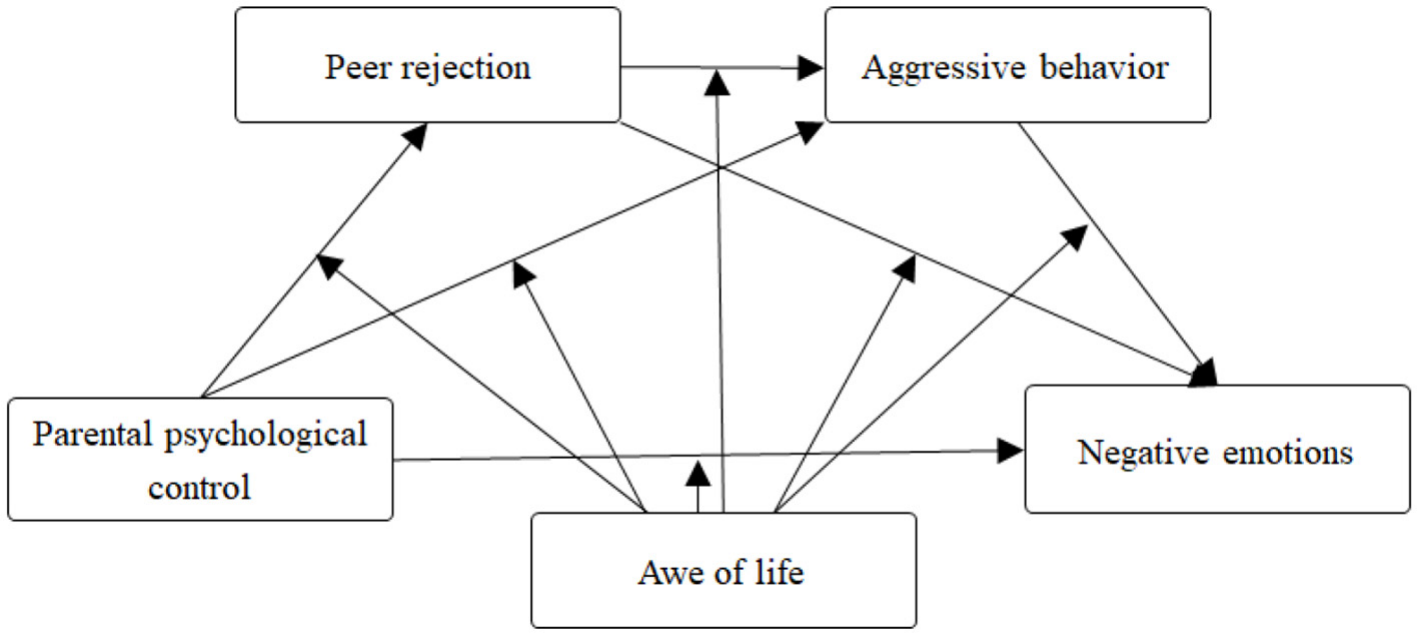
The hypothesized research model. Awe of life functions as a moderator across all pathways in the serial mediation model.

Based on the preceding analyses, this study proposes the following research hypotheses and theoretical model (Figure 1):

H1: Peer rejection mediates the relationship between parental psychological control and negative emotions.
H2: Aggressive behavior mediates the relationship between parental psychological control and negative emotions.
H3: Peer rejection and aggressive behavior form a serial mediating pathway in the association between parental psychological control and negative emotions.
H4: Awe of life moderates the association between parental psychological control and negative emotions.

## 2 Materials and methods

### 2.1 Participants and procedure

Using convenience sampling, our study recruited fifth and sixth graders from four elementary schools as participants in Anhui Province, China. Following administrative approval, class-wide sampling was implemented during information technology classes through standardized distribution of questionnaire links, with participants completing the assessments via computer terminals. Prior to data collection, informed consent was obtained from all participants and their guardians. Questionnaires were collected anonymously, with all responses maintained under strict confidentiality protocols. Under standardized administration by trained instructors using uniform protocols, 1,218 questionnaires were collected. After excluding invalid cases due to either implausibly short response times or detectable response patterns, 1,163 valid responses were retained, yielding a validity rate of 95.48%. The final sample consisted of 640 males (55.03%) and 523 females (44.97%), including 459 fifth-grade students (39.47%) and 704 sixth-grade students (60.53%). Participants' average age was 11.28 ± 0.80 years (M ± SD).

### 2.2 Measures

#### 2.2.1 Parents psychological control

The Parental Psychological Control Scale, developed by (Wang et al. 2007), consists of 18 items measuring three dimensions: guilt induction, love withdrawal, and authority assertion. Responses were recorded on a 5-point Likert scale (1 = not at all true; 5 = very true), with higher scores indicating greater levels of parental psychological control. The scale demonstrates good reliability and validity in Chinese adolescent populations (Li and Gu, 2023). In the current study, Cronbach's α coefficients were 0.93 for the full scale, 0.89 for guilt induction, 0.84 for love withdrawal, and 0.79 for authority assertion. The results of the confirmatory factor analysis supported the good structural validity of the scale, as evidenced by the following fit indices: χ^2^/df = 2.866, RMSEA = 0.046, CFI = 0.953, NFI = 0.947, IFI = 0.944.

#### 2.2.2 Peer rejection

The Peer Rejection Scale, originally developed by (Thau et al. 2015) and subsequently adapted by (Xu and Niu 2019), was administered, comprising 6 items. Using a 5-point Likert scale (1 = strongly disagree; 5 = strongly agree) where higher scores indicate greater peer rejection, this measure demonstrates good reliability and validity in Chinese adolescent populations (Dou et al., 2023), with a Cronbach's α of 0.93 in the current study. The results of the confirmatory factor analysis supported the good structural validity of the scale, as evidenced by the following fit indices: χ^2^/df = 2.248, RMSEA = 0.052, CFI = 0.968, NFI = 0.954, IFI = 0.953.

#### 2.2.3 Aggressive behavior

The Aggression Scale, originally developed by (Buss and Perry 1992) and subsequently adapted and abbreviated by (Liu et al. 2009), comprises 20 items measuring four dimensions: physical aggression, anger, hostility, and displaced aggression. Using a 5-point Likert scale (1 = strongly disagree; 5 = strongly agree) with higher scores indicating greater aggression levels, this measure demonstrates good reliability and validity in Chinese adolescent populations (Zou et al., 2024). In the current study, Cronbach's α coefficients were 0.92 for the full scale, 0.74 for physical aggression, 0.75 for anger, 0.77 for hostility, and 0.76 for displaced aggression. The results of the confirmatory factor analysis supported the good structural validity of the scale, as evidenced by the following fit indices: χ^2^/df = 3.194, RMSEA = 0.041, CFI = 0.964, NFI = 0.961, IFI = 0.957.

#### 2.2.4 Depression, anxiety and stress

The Depression Anxiety Stress Scales-21 (DASS-21), originally developed by (Lovibond and Lovibond 1995) and culturally adapted by (Wang et al. 2016), comprises 21 items measuring three dimensions (depression, anxiety and stress) with 7 items per subscale. Responses were recorded on a 4-point Likert scale (0 = did not apply to me at all; 3 = applied to me very much), with higher scores indicating more intense emotional experiences. The scale demonstrates good reliability and validity in Chinese adolescent populations (Chen et al., 2023), serving as an effective screening tool for general psychological distress. In the current study, Cronbach's α coefficients were 0.95 for the full scale, 0.87 for depression, 0.86 for anxiety, and 0.87 for stress. The results of the confirmatory factor analysis supported the good structural validity of the scale, as evidenced by the following fit indices: χ^2^/df = 2.864, RMSEA = 0.034, CFI = 0.969, NFI = 0.961, IFI = 0.953.

#### 2.2.5 Awe of life

The Adolescent Awe of Life Scale developed by (Lin et al. 2024), consists of 12 items assessing three dimensions: respect for life, fear of death, and transcendence of meaning of life. Responses were recorded on a 6-point Likert scale (1 = strongly disagree; 6 = strongly agree) with higher scores indicating greater levels of awe of life. The scale demonstrates good reliability and validity in Chinese adolescent populations, with Cronbach's α coefficients of 0.82 for the full scale, 0.83 for respect for life, 0.78 for fear of death, and 0.79 for transcendence of meaning of life. The results of the confirmatory factor analysis supported the good structural validity of the scale, as evidenced by the following fit indices: χ^2^/df =4.016, RMSEA = 0.065, CFI = 0.923, NFI = 0.917, IFI = 0.905.

### 2.3 Data analysis

Statistical analyses were conducted using SPSS 26.0, including descriptive statistics and Pearson correlation analyses. Conduct confirmatory factor analysis (CFA) on the scales using Amos 24.0. Based on correlational findings and theoretical hypotheses, linear regression modeling was employed to examine the relationship between parental psychological control and negative emotions. Data analyses were performed using Hayes' PROCESS macro (v4.1) in SPSS. Model 6 was employed to assess the serial mediating pathways linking parental psychological control to negative emotions through peer rejection and aggressive behavior, while Model 92 tested the moderating role of awe of life. Statistical significance was assessed via the bias-corrected percentile bootstrap approach with 95% confidence intervals (5,000 resamples), with associations deemed significant if the interval excluded zero.

## 3 Results

### 3.1 Common method bias testing

Common method bias was assessed through Harman's single-factor test by conducting an unrotated exploratory factor analysis, which yielded 11 factors with eigenvalues > 1, where the first factor accounted for 32.33% of the variance, below the 40% threshold (Podsakoff et al., 2003). Thus, common method bias in this study falls within acceptable levels.

### 3.2 Descriptive analysis

Pearson correlation analyses (Table 1) revealed significant positive associations among parental psychological control, peer rejection, aggressive behavior, and negative emotions (*r* = 0.489 ~ 0.738, all *p*s < 0.01). Awe of life showed significant negative correlations with parental psychological control, peer rejection, aggressive behavior, and negative emotions (*r* = −0.320 ~ −0.169, all *p*s < 0.01). These results provide preliminary support for testing mediation pathways. Additionally, given the significant positive associations between (a) gender and peer rejection, and (b) age and aggressive behavior, both variables were included as covariates in subsequent analyses.

**Table 1:** Participants' characteristics and correlation matrix.

**Variables**	**1**	**2**	**3**	**4**	**5**	**6**	**M**	**SD**
1. Gender	-						1.45	0.50
2. Age	−0.030	-					11.28	0.80
3. Parents psychological control	−0.025	0.026	-				43.75	16.43
4. Peer rejection	0.091^**^	0.028	0.498^**^	-			12.24	6.43
5. Aggressive behavior	0.015	0.076^**^	0.551^**^	0.680^**^	-		45.52	16.01
6. Awe of life	−0.065^*^	−0.071^*^	−0.169^**^	−0.246^**^	−0.239^**^	-	51.16	11.15
7. Negative emotions	0.056	0.042	0.566^**^	0.680^**^	0.738^**^	−0.320^**^	18.33	14.54

Gender: male = 1, female = 2; ^*^p < 0.05, ^**^p < 0.01.

### 3.3 Mediation model testing

Mediation analyses were performed using Model 6 of the PROCESS macro (v4.1) in SPSS to examine the intermediary mechanisms between parental psychological control and negative emotions (Table 2). Multiple regression analyses indicated a significant main association between parental psychological control and negative emotions (β = 0.567, *p* < 0.001). After incorporating the mediators (peer rejection and aggressive behavior), the direct association substantially weakened but remained significant (β = 0.179, *p* < 0.001).

**Table 2:** Testing the serial mediation model.

**Model**	**DV**	**IV**	**β**	***t***	***R^2^***	***F***
1	NE	Gender	0.071	2.934^**^	0.326	186.629^***^
		Age	0.030	1.229		
		PPC	0.567	23.477^***^		
2	PR	Gender	0.104	4.108^***^	0.259	131.190^***^
		Age	0.018	0.699		
		PPC	0.500	19.777^***^		
3	AB	Gender	−0.026	−1.254	0.525	320.611^***^
		Age	0.053	2.615^**^		
		PPC	0.280	11.942^***^		
		PR	0.541	23.003^***^		
4	NE	Gender	0.027	1.504	0.625	386.024^***^
		Age	−0.003	−0.175		
		PPC	0.179	8.092^***^		
		PR	0.288	11.405^***^		
		AB	0.443	16.964^***^		

All variables were standardized before being entered into the model. DV = dependent variable; IV = independent variable; PPC = parental psychological control; PR = peer rejection; AB = aggressive behavior; NE = negative emotions. Model 1 represents the total association between PPC and NE; Model 2 examines the association of PPC with PR; Model 3 assesses the associations of both PPC and PR with AB; Model 4 tests the associations among PPC, PR, AB and NE. ^**^p < 0.01, ^***^p < 0.001.

Bias-corrected percentile bootstrap analyses revealed significant indirect associations for all pathways, with 95% confidence intervals excluding zero (Table 3). Peer rejection and aggressive behavior serve as serial mediators in the association between parental psychological control and negative emotions. Additional analyses confirmed the significance of Indirect Path 1 (parental psychological control → peer rejection → negative emotions: β = 0.144, 95% CI (0.108, 0.183), which supported H1 and explained 25.40% of the total association. Indirect Path 2 (parental psychological control → aggressive behavior → negative emotions: β = 0.124, 95% CI (0.092, 0.159) was significant, supporting H2 and explaining 21.87% of the total association. Indirect Path 3 (parental psychological control → peer rejection → aggressive behavior → negative emotions: β = 0.120, 95% CI (0.096, 0.146) demonstrated significant serial mediation, supporting H3 and explaining 21.16% of the total association.

**Table 3:** Decomposition of total, direct and indirect associations.

**Paths**	**Values**	**Boot SE**	**95% CI**	**Ratio**
1. PPC → PR → NE	0.144	0.019	(0.108, 0.183)	25.40%
2. PPC → AB → NE	0.124	0.017	(0.092, 0.159)	21.87%
3. PPC → PR → AB → NE	0.120	0.012	(0.096, 0.146)	21.16%
Total indirect association	0.388	0.023	(0.346, 0.430)	68.43%
Direct association	0.179	0.022	(0.135, 0.222)	31.57%
Total association	0.567	0.024	(0.519, 0.614)	

PPC = parental psychological control; PR = peer rejection; AB = aggressive behavior; NE = negative emotions.

The direct and indirect associations accounted for 31.57% and 68.43% of the total association, respectively, indicating that indirect pathways exerted over twice the magnitude of direct associations in the link between parental psychological control and negative emotions, and peer rejection showed a particularly strong association.

### 3.4 Moderated model testing

**Figure 2:**
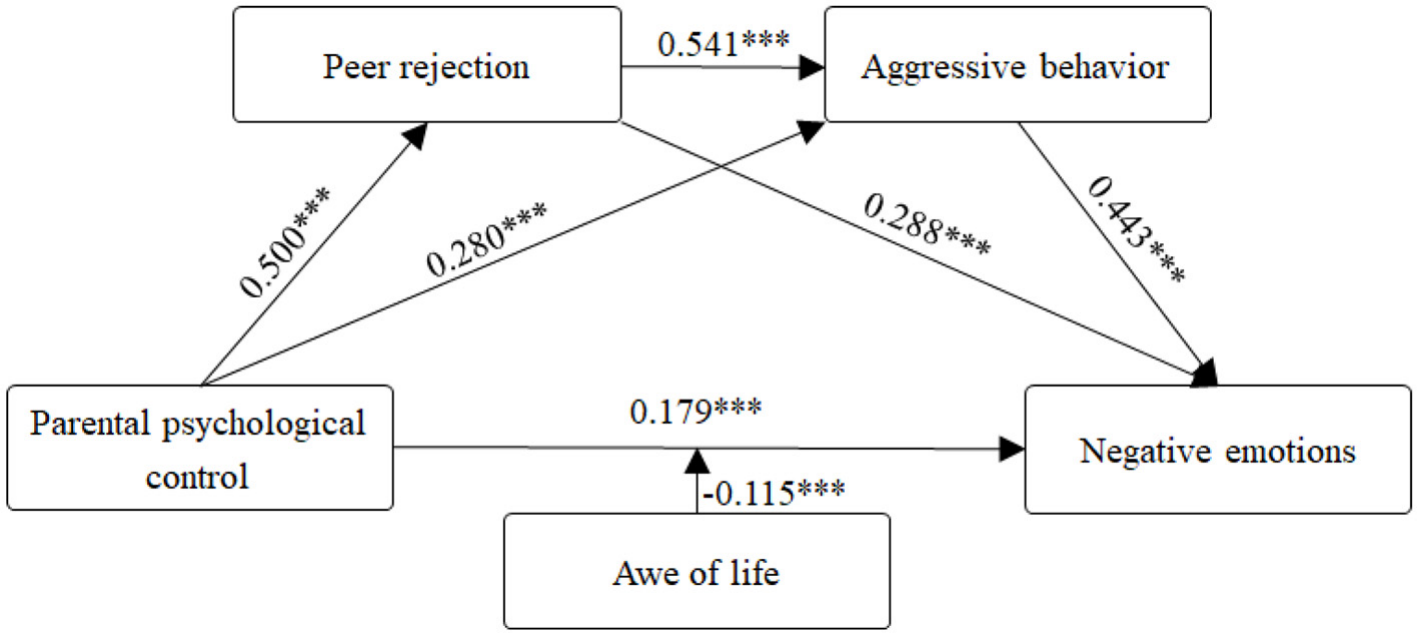
The standardized estimate values of the final model. ^***^p < 0.001.

The direct association between parental psychological control and negative emotions was significant, supporting the establishment of a moderated serial mediation model (Igartua and Hayes, 2021). To examine the moderation of awe of life, we conducted analyses using Model 92 of the PROCESS macro (v4.1) in SPSS. The interaction term between parental psychological control and awe of life showed a significant association with negative emotions (β = −0.060, *t* = −2.988, *p* < 0.01). In contrast, no other interaction terms involving awe of life reached statistical significance (Table 4). These results demonstrate that awe of life significantly moderated the direct association between parental psychological control and negative emotions, whereas its moderating influences on all indirect pathways were nonsignificant (Figure 2), providing partial support for H4.

**Table 4:** Testing the moderated mediation association.

**DV**	**IV**	**β**	***t***	***R^2^***	***F***
PR	Gender	0.182	3.614^***^	0.285	92.086^***^
	Age	0.010	0.304		
	PPC	0.470	18.491^***^		
	AF	−0.155	−6.014^***^		
	PPC × AF	−0.026	−1.190		
AB	Gender	−0.054	−1.319	0.530	185.978^***^
	Age	0.063	2.466^*^		
	PPC	0.276	11.741^***^		
	PR	0.527	22.054^***^		
	AF	−0.057	−2.671^**^		
	PPC × AF	0.020	0.922		
	PR × AF	−0.032	−1.511		
NE	Gender	0.034	0.950	0.642	229.499^***^
	Age	−0.011	−0.512		
	PPC	0.169	7.798^***^		
	PR	0.269	10.823^***^		
	AB	0.430	16.716^***^		
	AF	−0.115	−6.098^***^		
	PPC × AF	−0.060	−2.988^**^		
	PR × AF	0.002	0.091		
	AB × AF	0.020	0.812		

DV = dependent variable; IV = independent variable; PPC = parental psychological control; PR = peer rejection; AB = aggressive behavior; AF = awe of life; NE = negative emotions; ^*^p < 0.05, ^**^p < 0.01, ^***^p < 0.001.

**Figure 3:**
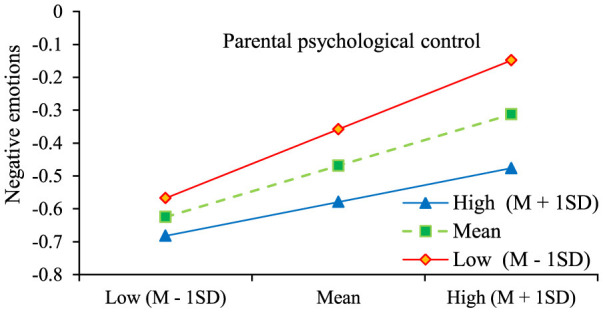
Moderating role of awe of life in the direct path of parental psychological control and negative emotions. Higher levels of awe of life correspond to stronger buffering capacity.

Further analysis shows that the association between parental psychological control and negative emotions was significantly moderated by awe of life across all three tested levels, with association strengths demonstrating a decreasing trend, indicating a buffering role of awe of life in this association. Simple slope analyses (Figure 3) revealed that stronger associations between parental psychological control and negative emotions under low awe of life (M-1SD), whereas high levels (M + 1SD) effectively buffered this adverse influence (Table 5).

**Table 5:** Indirect values at different levels of awe of life.

**Level**	**Values**	**SE**	***t***	**95% CI**
M-1SD	0.229	0.028	8.052^***^	(0.173, 0.285)
M	0.169	0.022	7.798^***^	(0.127, 0.212)
M+1SD	0.110	0.031	3.589^***^	(0.050, 0.169)

^***^p < 0.001. The moderating roles demonstrate statistical significance across all three tested levels.

## 4 Discussion

Grounded in the Ecological Systems Theory (Bronfenbrenner, 1977) and Cumulative Risk Model (Evans et al., 2013), this study integrates microsystem-level family factors, peer factors, and individual behaviors to examine their collective associations with negative emotions. The results demonstrated that parental psychological control is directly associated with negative emotions, while also exerting influence through both the independent mediation of peer rejection and aggressive behavior, as well as their serial mediation pathway. The awe of life exerts a significant moderation on the direct pathway, attenuating the link between parental psychological control and negative emotions.

### 4.1 The mediating role of peer rejection and aggressive behavior

The total association between parental psychological control and negative emotions is 0.567, with a mediating value of 0.388, accounting for 68.43% of the total association, demonstrating significant implications for the mental health in early adolescence. (Erikson 1968) theory of psychosocial development identifies adolescence as a critical period for ego identity formation. Parental psychological control, defined as an intrusive parenting behavior that compromises emotional autonomy (Steinberg et al., 1992), has been shown to significantly impair the development of psychological autonomy and identity consolidation (Barber and Harmon, 2002). This parenting approach diminishes children's perceived control and internal locus of control, undermines their emotional functioning (Wang et al., 2007), fosters helplessness development, and ultimately leads to internalizing problems. These associations collectively contribute to internalizing problems (Chyung et al., 2021; Garber and Flynn, 2001), including elevated negative emotions, which aligns with prior findings (Loparo et al., 2023; Lee et al., 2015).

Additionally, peer relationships play an extremely important role in early adolescence. According to Ecological Systems Theory (Bronfenbrenner, 1977), family and peers, as two crucial subsystems influencing adolescent development, do not evolve independently but are interrelated, whereby risk factors in one subsystem heighten vulnerability to risk factors in the other (Hong et al., 2022).

Children exposed to high levels of parental psychological control tend to develop maladaptive interpersonal strategies, leading to peer rejection (Hong et al., 2022), which in turn elevates the risk of anxiety and depression (Li and Jiang, 2018; Wang et al., 2019; Zhang et al., 2018). Although some studies posit peer rejection as a consequence rather than a cause of internalizing symptoms (Vaillancourt et al., 2013), the current study supports the temporal precedence of peer rejection over the development of internalizing symptoms (Healy et al., 2024).

According to the Temporal Need-Threat Model (Williams, 2009), thwarted needs drive individuals to enact specific behaviors directed toward need satisfaction or compensation, with aggressive behavior constituting a prevalent response pattern. Excluded individuals resort to aggression to regain relational control or dominance, thereby compensating for impaired fundamental needs (Reiter-Scheidl et al., 2018). This finding substantiates and reinforces previous research demonstrating that peer-rejected children may develop heightened hostile intent, consequently eliciting aggressive behaviors (Dodge et al., 2003; Reijntjes et al., 2011).

Our study reveals a serial mediation pathway wherein parental psychological control influences aggressive behavior through peer rejection, confirming the “risk cascade” hypothesis of Cumulative Risk Models. Specifically, parental psychological control not only directly erodes adolescent autonomy (Self-Determination Theory; Deci and Ryan, 2000), but also through social learning mechanisms (Bandura, 1977), facilitates the acquisition of coercive interpersonal strategies (e.g., emotional manipulation), leading to peer rejection. Such rejection then jeopardizes fundamental belongingness needs (Need-Threat Model; Williams, 2009), which predisposes individuals to aggressive behaviors as compensatory mechanisms (e.g., reestablishing perceived control via aggression), thereby establishing a cumulative risk sequence of “control → rejection → aggression → emotional dysregulation”. These findings corroborate (Evans et al. 2013) proposition of “Nonlinear Additive Effects of Multiple Risk Factors”, illustrating how familial, peer, and behavioral risk factors exacerbate mental health problems via progressive cascades. Among the three indirect pathways, peer rejection showed the strongest mediating role. This pattern aligns with developmental research indicating heightened sensitivity to peer relationships during early adolescence, when needs for group belongingness and friendship become particularly pronounced (Beeson et al., 2020).

### 4.2 The moderating role of awe of life

Partially supporting H4, awe of life significantly moderated the direct path from parental psychological control to negative emotions, but not the indirect paths.

The moderating role indicates that awe of life buffers the direct association between parental psychological control and negative emotions. Individuals with high levels of awe of life exhibit significantly lower negative emotions when exposed to psychological control, whereas those with low awe of life demonstrate heightened vulnerability to its adverse associations.

Emotion Regulation Theory posits that emotions result from the operations of valuation systems (Gross, 2015). In the direct path, awe of life functions as a “second-level” valuation system, assessing and modulating the “first-level” valuation system that generates negative emotions. Since awe of life prompts individuals to reexamine the events causing negative emotions from a broader, more macroscopic perspective of life (Monroy and Keltner, 2023), it facilitates adjustment of the cognition and evaluation of these events. On the one hand, when encountering negative emotion-eliciting situations, awe of life directs attention to life's profound value and beauty, enabling individuals to extract positive significance from adversity (Liao et al., 2024). The process of meaning-making promotes cognitive reappraisal, effectively downregulating negative emotions. On the other hand, awe motivates the adoption of proactive emotional goals, predisposing individuals to prioritize positive emotional experiences. Through this dual pathway, awe weakens the association with negative emotions while concurrently enhancing subjective wellbeing and life satisfaction (Jin, 2021).

The indirect pathway, encompassing peer and individual behavioral factors, involves complex emotion-related processes wherein the moderating role of awe of life may be obscured or attenuated by other mediating variables, thereby resulting in non-significant moderation.

### 4.3 Implications and limitations

The findings provide valuable insights for contemporary family education and school education practices in China.

Despite China's implementation of the three-child policy, the declining birth rate remains an indisputable fact. Smaller family size elevates parental expectations for children (Milovanska-Farrington, 2022), heightening parental anxiety. This heightened anxiety may trigger unconscious exertion of psychological control when children exhibit behavioral deviations. Furthermore, traditional Chinese Confucian culture emphasizes parental authority and filial piety, where strong control is often interpreted as an expression of parental love (Li et al., 2025). In collectivist cultural contexts, parents perceive their children as extensions of their own self-worth. To enhance parental status and image, they may therefore unconsciously intensify control over their children, with the aim of motivating children toward higher achievements. However, intensive psychological control may trigger cumulative negative emotions and heighten vulnerability to suicidal ideation and behaviors (Breslin et al., 2020), ultimately undermining healthy child development (Ma and Zhang, 2022).

As (Grolnick 2003) noted, “Well-meaning parenting backfires”. Parents should provide greater support for children's autonomy and initiative (Lee et al., 2015), maintain openness to unpredictability (Wolbert et al., 2018), and ensure students feel genuinely respected, an approach that fosters holistic psychological and physical development.

Schools should acknowledge the significant relevance of peer rejection and aggressive behaviors to adolescent mental health while leveraging their unique resource advantages to foster psychological wellbeing (Yu et al., 2022). Specifically, in the context of the “Double Reduction” policy, educational institutions should design innovative curricula targeting social-emotional competencies and stress management techniques, systematically integrating social skills training into core education to better mitigate peer rejection. Concurrently, teachers should monitor students' emotional and behavioral patterns, developing specialized competence in early identification of mental health risks (O'Farrell et al., 2022). Furthermore, implementing physical activities and group experiential programs while strengthening life education enhances students' awe of life, effectively mitigating emotional problems and improving psychological wellbeing (Tu, 2020; Moeijes et al., 2018).

This study has several limitations. First, although gender and age were controlled for in the current study, potential confounding factors such as household income and parental education level were not included, which may compromise the ecological validity of the findings. Future research should incorporate multilevel data to verify the model's generalizability. Second, this study employed self-report questionnaires, with data collected solely from students' subjective reports. This approach may introduce issues such as common method bias and social desirability bias. Future research should integrate behavioral observations, physiological indicators (e.g., heart rate, skin conductance response), and multi-informant reports (e.g., from teachers, peers, parents) to further elucidate the mechanisms underlying adolescents' negative emotions. Third, the cross-sectional design with samples drawn exclusively from four elementary schools in Anhui Province precludes causal inferences. Future investigations should employ longitudinal or experimental designs to conduct cross-regional and cross-cultural studies, thereby enhancing the generalizability of findings.

## 5 Conclusion

This study investigated the association between parental psychological control and negative emotions, clarifying the underlying mechanisms by examining the mediating roles of peer rejection and aggressive behaviors, as well as the moderating role of awe of life. This study reveals a risk-cascade mechanism of “parental control → peer rejection → aggressive behavior” and the buffering protective role of awe of life. It establishes the first multi-risk–protection model tailored to Chinese early adolescents, closing a critical gap in developmental research on this age period and providing precise intervention targets for the early prevention of adolescent emotional problems. Our study highlights that reducing parental psychological control, promoting healthy peer relationships, and nurturing children's awe of life have significant implications for predicting and preventing mental health risks among early adolescents.

## Data Availability

The original contributions presented in the study are included in the article/Supplementary material, further inquiries can be directed to the corresponding author.
